# Cubic chemically ordered FeRh and FeCo nanomagnets prepared by mass-selected low-energy cluster-beam deposition: a comparative study

**DOI:** 10.3762/bjnano.7.177

**Published:** 2016-11-28

**Authors:** Veronique Dupuis, Anthony Robert, Arnaud Hillion, Ghassan Khadra, Nils Blanc, Damien Le Roy, Florent Tournus, Clement Albin, Olivier Boisron, Alexandre Tamion

**Affiliations:** 1 Institut Lumière Matière, UMR5306 Université Lyon 1-CNRS, Université de Lyon F-69622 Villeurbanne cedex, France; 2 Institut Jean Lamour, UMR7198, Université H. Poincarré-CNRS, F-5406 Vandoeuvre les Nancy, France; 3 Interfaces, Confinement, Matériaux et Nanostructures, UMR7374 Université d'Orléans-CNRS, F-45071 Orléans cedex, France; 4 Université Grenoble Alpes, Inst NEEL, F-38000 Grenoble, France; 5 CNRS, Inst NEEL, F-38000 Grenoble, France

**Keywords:** bimetallic nanoparticles, magnetic moment and anisotropy, metamagnetic transition

## Abstract

Near the point of equiatomic composition, both FeRh and FeCo bulk alloys exhibit a CsCl-type (B2) chemically ordered phase that is related to specific magnetic properties, namely a metamagnetic anti-ferromagnetic/ferromagnetic transition near room temperature for FeRh and a huge magnetic moment for the FeCo soft alloy. In this paper, we present the magnetic and structural properties of nanoparticles of less than 5 nm diameter embedded in an inert carbon matrix prepared by mass-selected low-energy cluster-beam deposition technique. We obtained a CsCl-type (B2) chemically ordered phase for annealed nanoalloys. Using different experimental measurements, we show how decreasing the size affects the magnetic properties. FeRh nanoparticles keep the ferromagnetic order at low temperature due to surface relaxation affecting the cell parameter. In the case of FeCo clusters, the environment drastically affects the intrinsic properties of this system by reducing the magnetization in comparison to the bulk.

## Introduction

Magnetic bimetallic nanoparticles (NPs) are very attractive systems not only from a fundamental point of view but also because of their various areas of use [1–2]. In particular, the binary phase diagrams of bulk materials of iron and transition metals show a wide range of different properties, in particular magnetic properties [3].

**Figure 1:**
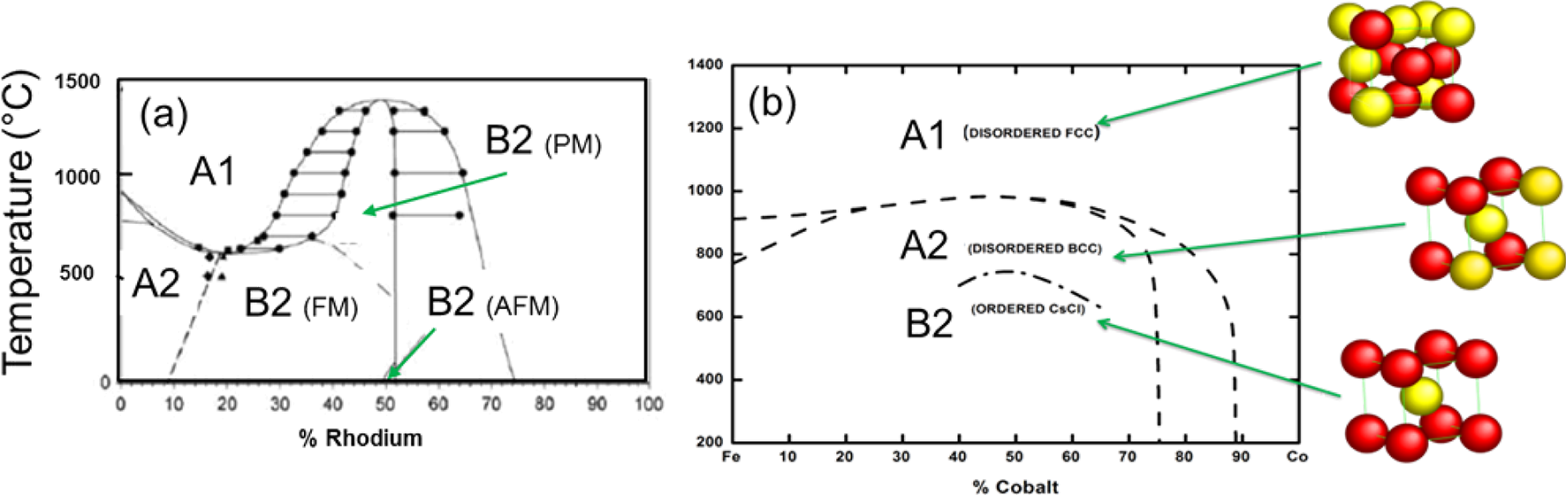
(a) Bulk magnetic FeRh phase diagram. Notice that metamagnetic transitions in bulk B2 FeRh are expected from the anti-ferromagnetic (AFM) to the ferromagnetic (FM) state at 96 °C and to the paramagnetic (PM) state at 380 °C. (b) Bulk FeCo phase diagram showing the different chemical and crystallographic orders for equiatomic concentration (adapted from [4]).

Interestingly, near the point of equiatomic composition, both FeRh and FeCo bulk alloys present a CsCl-type (B2) chemically ordered phase at room temperature (Figure 1) with the competition between several magnetic orderings for FeRh and a huge magnetic moment for soft FeCo according to the Slater–Pauling graph.

It is interesting to examine the chemical and magnetic ordering at the nanoscale in both FeRh and FeCo alloys for their potential applications in heat-assisted magnetic recording and spintronics [5–6]. Several routes have been developed to produce such bimetallic nanoparticles during which annealing is generally necessary to give the nanoalloy enough energy to reach its thermodynamic equilibrium [1]. Considering chemical syntheses, Jia et al. [7] have shown that the coalescence of initially 4–5 nm FeRh NPs to structures of 20 nm in diameter after annealing is necessary to observe AF–FM transition in chemically ordered NPs. Recently, a strong correlation between morphology and magnetism has also been predicted from theoretical density functional theory calculations in agreement with experiments on FeRh nanoparticles prepared by co-sputtering [8]. In addition, for FeCo nanoparticles generated in the gas phase by means of an arc cluster ion source, a wide distribution of magnetic energy barriers has been obtained in a mass-filtered ensemble of particles with a mean diameter of 12 nm and size distribution lower than 15% [9].

In this paper, we present a comparative study performed on FeRh and FeCo nanocrystal assemblies prepared by mass-selected low-energy cluster-beam deposition (MS-LECBD) embedded in a carbon matrix. Notice that most of this work is based on the results of the PhD theses of A. Hillion [10] and G. Khadra [11] at Lyon, France. The structural and magnetic properties of as-prepared and annealed nanoalloys were investigated using various experimental techniques [4]. Here, we focus on anomalous X-rays diffraction (AXD) and X-ray magnetic circular dichroism (XMCD) performed by using well-adapted synchrotron radiation beamlines. We show how the competition between the stable bimetallic NPs structure and their chemical affinity with the environment affects their intrinsic magnetic properties compared to their bulk counterparts.

## Results

### Synthesis and structure

The clusters are synthetized in the gas phase in the low-energy cluster-beam deposition (LECBD) regime. Briefly, a pulsed laser beam is focused on a mixed equiatomic target while a continuous flow of helium allows the formation of the cluster beam. After isentropic expansion in vacuum, ionized species can be size-selected thanks to a quadupolar electrostatic deviator [4].Then, the mass-selected clusters are simultaneously co-deposited in an ultra-high vacuum (UHV) deposition chamber, with an independent atomic carbon beam. Notice that the strong asset of this experimental technique is the possibility to prepare nearly identical clusters in any matrix, with the possibility to vary the volume concentration of the magnetic phase.

**Figure 2:**
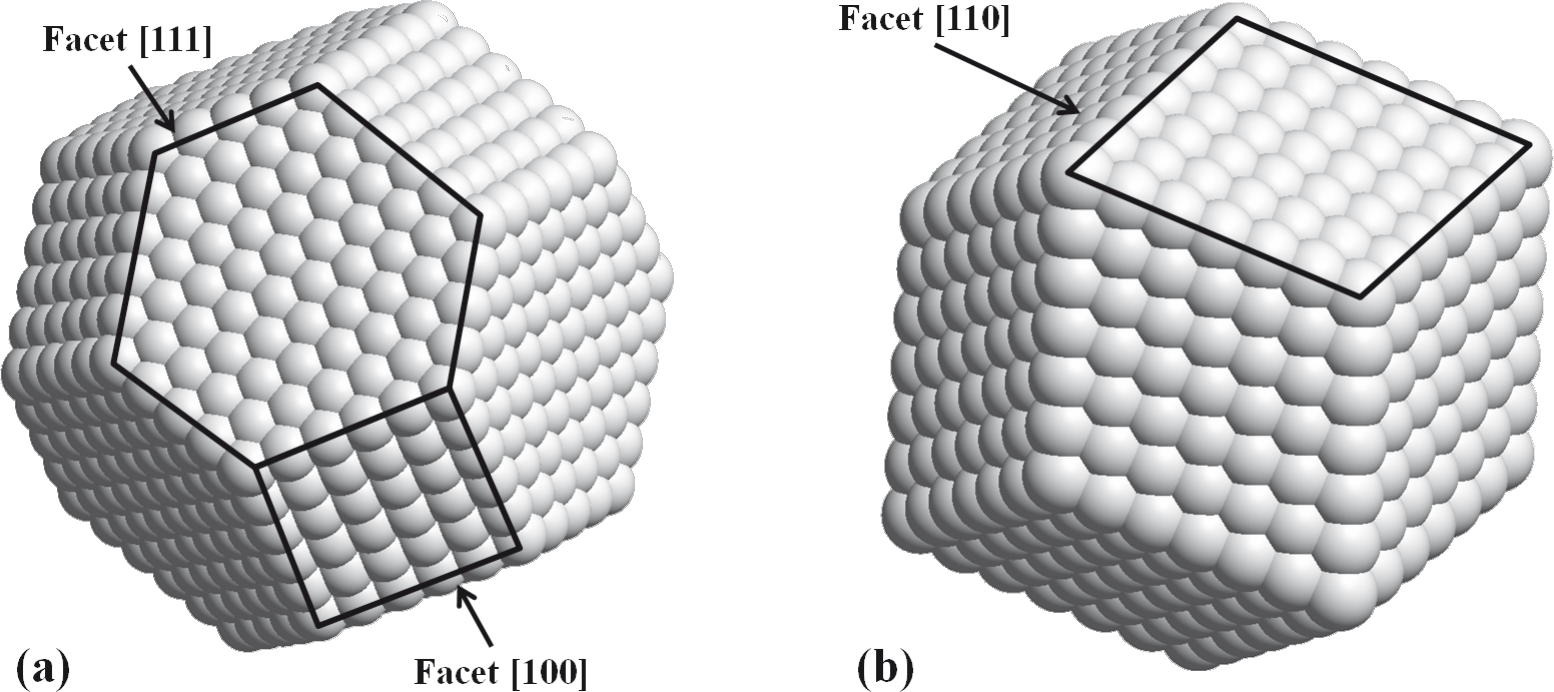
Stable shape for a face-centred cubic (fcc) truncated octahedron (a) and a body-centred cubic (bcc) rhombic dodecahedron (b) [11].

According to the Wulff construction [12], we have been able to systematically show that our clusters are nanocrystallized and well-faceted (Figure 2).

In order to avoid magnetic interactions among the NPs, the samples are prepared with a cluster concentration of less than 1 vol %. Notice that the amorphous carbon matrix is chosen to protect the sample from oxidation but also to allow vacuum high-temperature annealing and so to reach the equilibrium phase without coalescence of the NPs.

**Figure 3:**
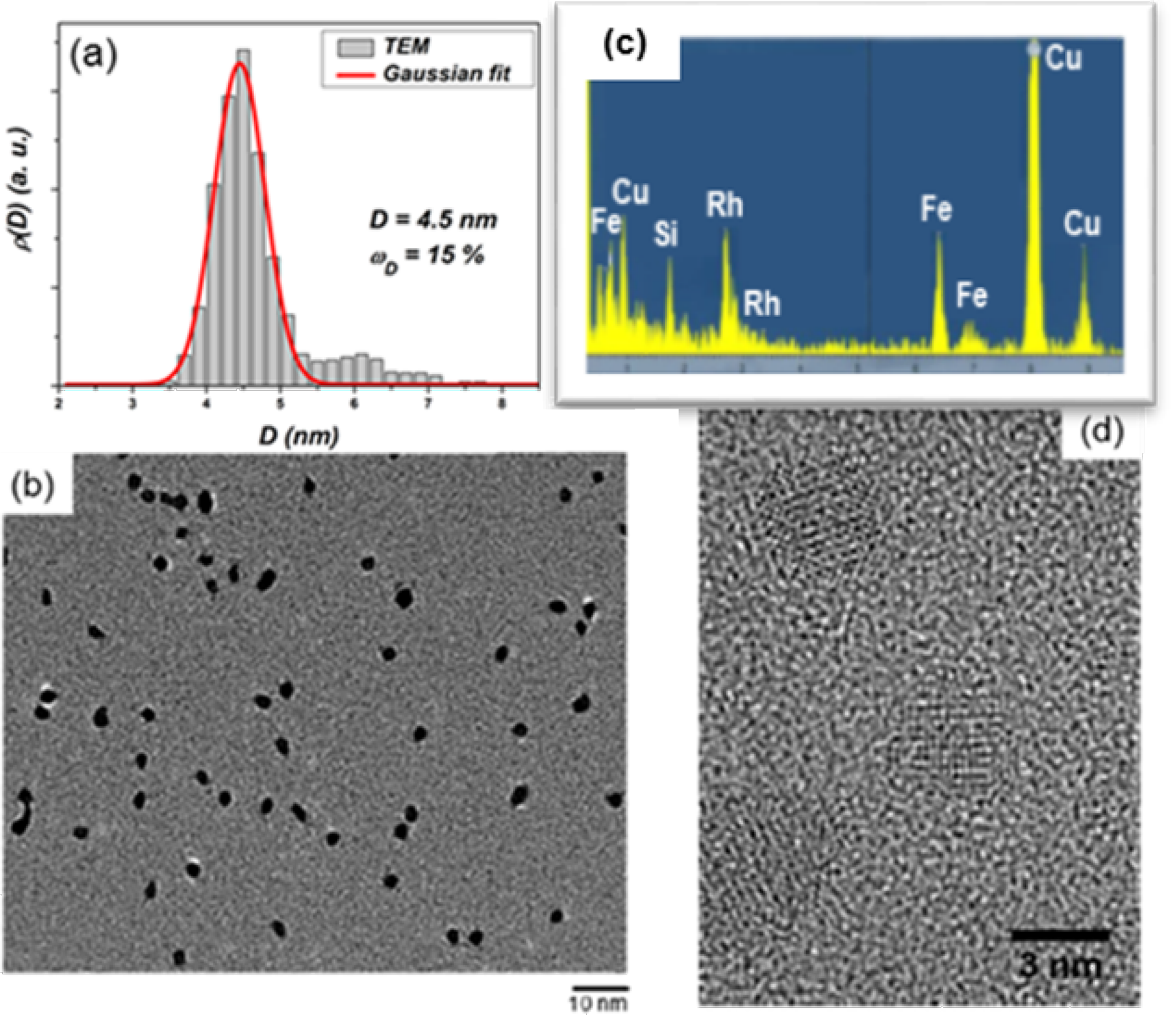
Size histogram (a), TEM observations (b) and corresponding EDX analysis (c) of annealed mass-selected FeRh nanoparticles with an average diameter of 4.5 nm. HRTEM images for annealed FeRh nanoparticles with 2 nm in diameter (d).

To characterise the structure of the clusters by transmission electron microscopy (TEM), we prepared discontinuous thin layers of NPs deposited on an carbon-coated grid and then capped it with a thin carbon film. We obtained nanoparticles with selected diameters from 2 to 5 nm, a Gaussian size distribution and a relative dispersion of around 0.15 (Figure 3).

In addition, several nanoparticles were analyzed using EDX (energy dispersive X-ray analysis) as shown in Figure 3c. In all cases, the EDX analysis showed no sign of oxidation of the nanoparticles and a roughly equiatomic composition for both FeRh and FeCo cluster samples conserved after 2 h annealing at 500 °C under ultra-high vacuum (UHV) conditions [10–11].

**Figure 4:**
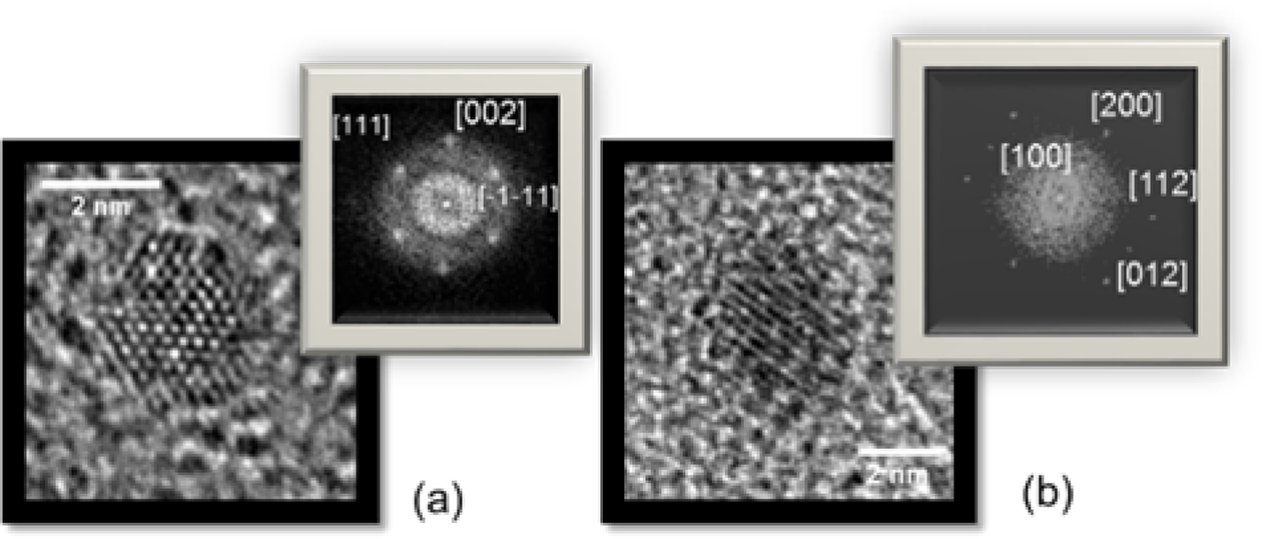
HRTEM images for as-prepared (a) and annealed (b) FeRh nanoparticles with their respective FFT corresponding to fcc (A1) (a) and CsCl-type (B2) phase (b). Figure 4a is a slightly changed reproduction from [13], copyright 2013 American Physical Society.

High-resolution TEM (HRTEM) observations on as-prepared FeRh samples revealed a clear fcc (A1) structure with no sign of chemical ordering (Figure 4a). In the Figure 3d and Figure 4b, chemically ordered CsCl-type (B2) nanomagnets have been clearly evidenced on annealed FeRh NPs as small as 2 nm in diameter.

Even though the CsCl (B2) structure is the equilibrium bulk structure for equimolar FeCo alloys, it was not possible to distinguish a chemical ordering by HRTEM for annealed NPs because the electron density contrast between iron and cobalt is too low.

Generally, conventional diffraction measurements are used to determine the degree of chemical ordering in the B2 phase for equiatomic AB alloys. The B2 structure can be seen as the imbrication of two simple cubic sub-lattices, each occupied by either A or B atoms (with A = Fe and B = Rh/Co for FeRh/FeCo). Indeed, the structure factor is expected to present two maxima at *f*_A_* + f*_B_ (main reflection, with even *h* + *k* + *l* value for the sum of Miller indices, as in bcc structures) and at *f*_A_ − *f*_B_ (secondary or superlattice reflection, only for B2 structures with odd *h* + *k* + *l* values related to the atomic number difference Δ*Z*_AB_ for X-rays and electrons). The supplementary reflections (100) and (012) are clearly visible on the FFT of Figure 4b for annealed FeRh (Δ*Z*_FeRh_ = 19). While in the case of FeCo alloy, the secondary minima are negligible due to the small value of the atomic contrast (Δ*Z*_FeCo_ = 1). Because the nuclear scattering factors for iron and cobalt differ considerably, neutron diffraction is generally preferred over X-ray diffraction to determine the degree of long-range order in FeCo alloy [14]. However, neutron diffraction is not applicable for the low quantities of matter in our samples.

**Figure 5:**
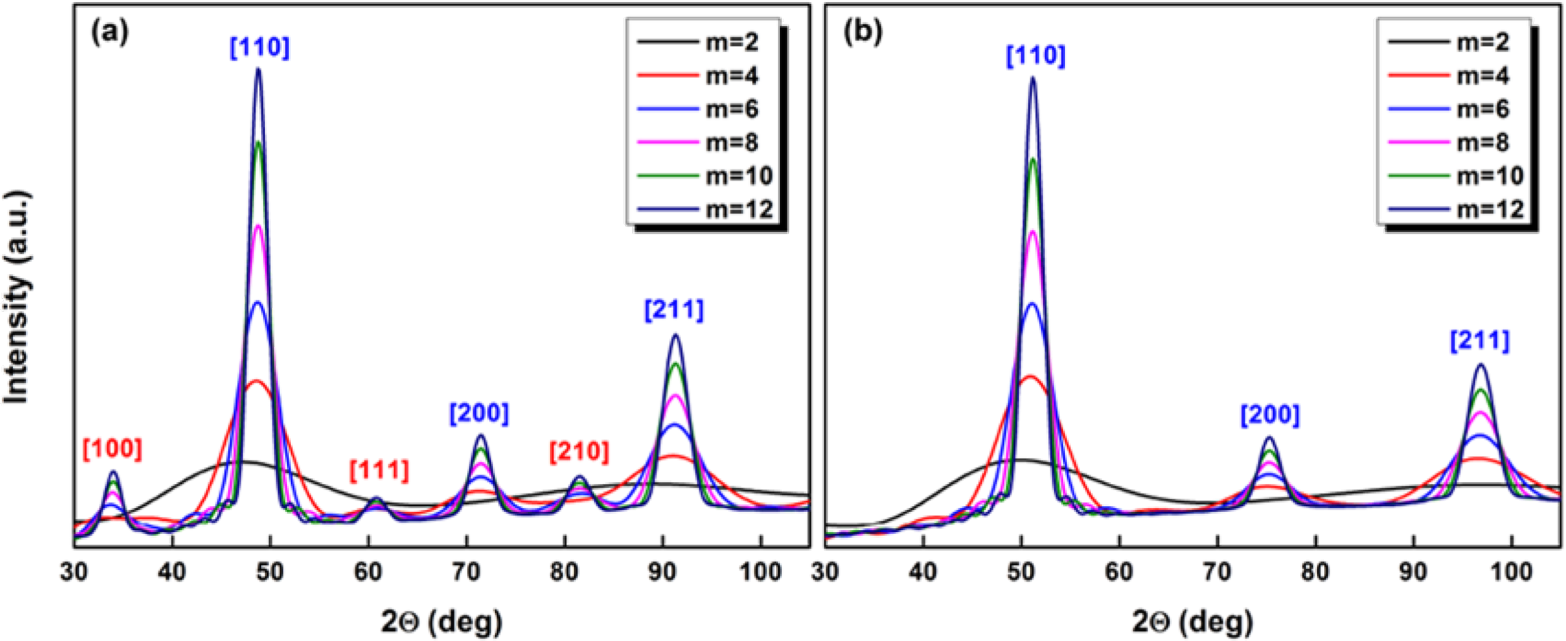
Simulated X-ray scattering curves for CsCl-type (B2) phase (a) FeRh and (b) FeCo nanoparticles with different sizes [11].

Moreover, at finite size we also have to take into account the shape factor that enlarges the Bragg–Dirac peak distribution expected only for infinite crystals. So for both systems, a the CsCl-type (B2) structure was assumed for the simulations by using the Debye formula as previously developed on mass-selected L1_0_ CoPt nanoparticles with truncated-octahedron shape, as shown in Figure 2a [15]. Figure 5 shows the simulated curves expected for the FeRh and FeCo assemblies of perfect rhombic dodecahedrons (as in Figure 2b) with different sizes governed by the number of atoms per edge *m* (*m* = 12 corresponds to nanoparticles with a size of around 5 nm) [11].

In the case of FeRh nanoparticles (Δ*Z*_FeRh_ = 19), the CsCl-type (B2) phase signature, in red in Figure 5a, should be identifiable in our range size for FeRh nanoparticles but progressively vanishes as the size decreases. While in the case of FeCo, it should be impossible to extract the superlattice reflection peaks for small nanoparticles up to *m* = 12, our largest size.

**Figure 6:**
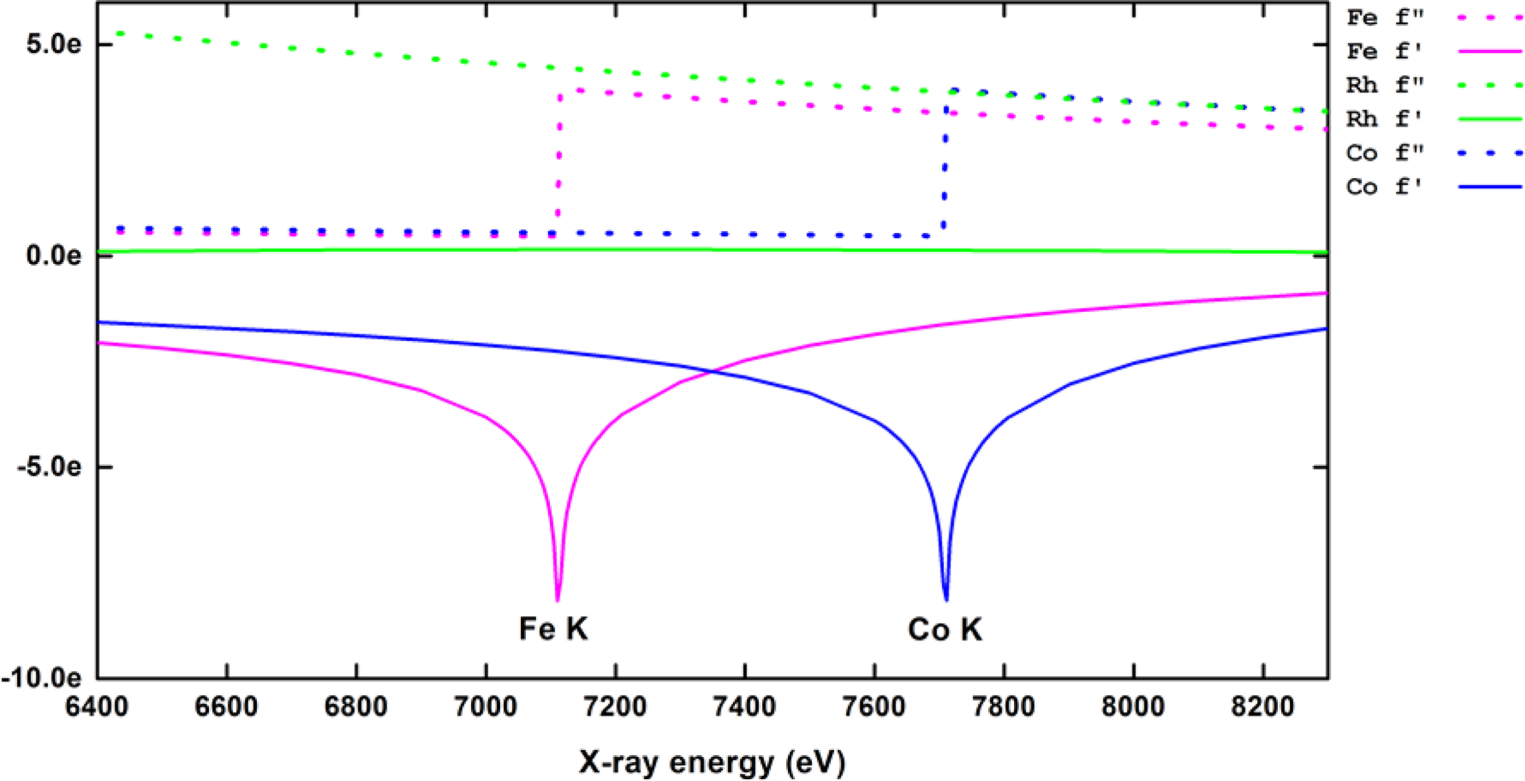
Anomalous scattering *f*′ and *f*″ coefficients as a function of photon energy for Fe, Co and Rh elements in the range of Fe and Co K edges.

To go a step further in the investigation of the chemical ordering in FeCo, we used anomalous X-ray diffraction (AXD) in order to experimentally overcome the low “*Z*-contrast” between Fe and Co. This was achieved by changing the X-ray wavelength (or photon energy) by using synchrotron radiation techniques, allowing for chemical selectivity and high photon flux. Indeed, for X-ray diffraction, the atomic scattering factor *f* is a complex number and can be written as follows: *f = f*_0_*+ f*′(λ) *+* i*f*″(λ)*,* where *f*_0_ ~ *Z* while *f*′ and *f*″ are wavelength-dependent especially around the absorption edge for heavy atoms [16]. From Figure 6, we found that a photon energy of 7.108 keV just before the Fe K absorption edge gave a maximum of anomalous contrast for the atomic scattering factor equal to nine for FeCo, larger than the atomic contrast (Δ*Z*_FeCo_ = 1).

**Figure 7:**
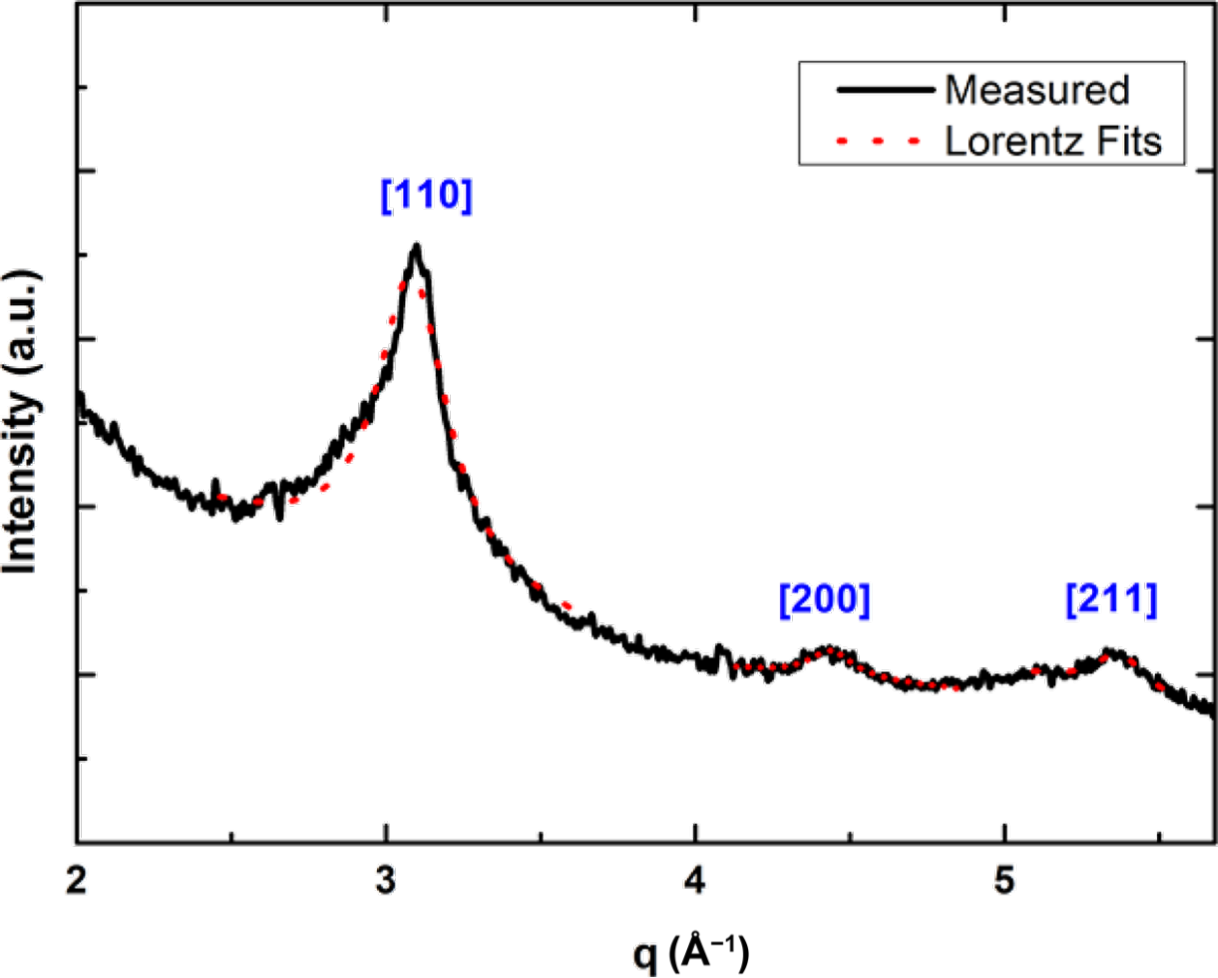
Measured X-ray scattering at 7.108 keV on annealed FeCo nanoparticles with 5 nm in diameter.

Thus, mass-selected 5 nm-FeCo nanoparticles were measured with AXD after annealing at 500 °C for 2 h. The measurements were performed on the BM02-D2am French CGR beamline at the ESRF (Grenoble, France) at an X-ray energy fixed at 7.108 keV. The incidence angle was optimized after calibrations so as to have a good compromise between low signal from the Si substrate and maximum intensity of the Bragg (110) peak. The Figure 7 shows the measured X-ray scattering where we can see three peaks which correspond to the main Bragg peaks common to the B2 and bcc structures.

These three peaks, showing the very good crystallinity of the annealed FeCo nanoparticles embedded in the carbon matrix, were isolated and fitted with a Lorentz-type function. Using the Debye–Scherrer equation [17], the size of the nanoparticles is estimated based on the width of the scattered peaks:


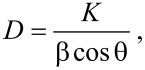


where *D* is the diameter of the nanoparticle, *K* is a dimensionless shape factor (approximated as *K* ≈ 0.9), λ is the X-ray wavelength, β is the full width at half maximum (FWHM) of the peak and θ is the Bragg angle. The corresponding values obtained for both the Lorentz fit and the obtained estimated diameters are presented in Table 1.

**Table 1:** Values obtained for the Scherrer diameter (*D*_Scherrer_) as well as the peak position and width for the X-ray scattering spectrum on annealed FeCo nanoparticles with 5 nm in diameter [11].

peak	2θ (°)	FWHM (°)	*D*_Scherrer_ (nm)

(110)	50.7	4.83	4.13
(200)	76.1	3.46	6.60
(211)	96.5	5.36	5.04

Averaging the diameter values obtained from the three peaks we obtain *D*_Scherrer_ = 5.25 nm, which is consistent with the results obtained from TEM microscopy for our mass-selected annealed FeCo nanoparticles. But again it was not possible to find evidence of any superstructure B2 peaks in our largest FeCo nanoparticles from AXD.

Therefore, we have performed a series of extended X-ray absorption fine structure (EXAFS) measurements on FeCo samples, at both Fe and Co K edges, to describe local order at each atomic site for different cluster sizes. We have had to develop a specific strategy, more complex than for our previous analysis in bimetallic clusters [18]. As shown in [11], the B2 phase is confirmed for the larger FeCo NPs while a detailed study will be published elsewhere. As a conclusion, the stable B2 chemical ordering is mainly conserved in both FeRh and FeCo nanoalloys. In the next section, we report on the magnetic behaviour of our nanoalloys.

### Magnetic characterization

First, the magnetic properties of Fe-based clusters embedded in a carbon matrix have been studied by superconducting quantum interference device (SQUID) magnetometry experiments and simulations [4,19–21].

**Figure 8:**
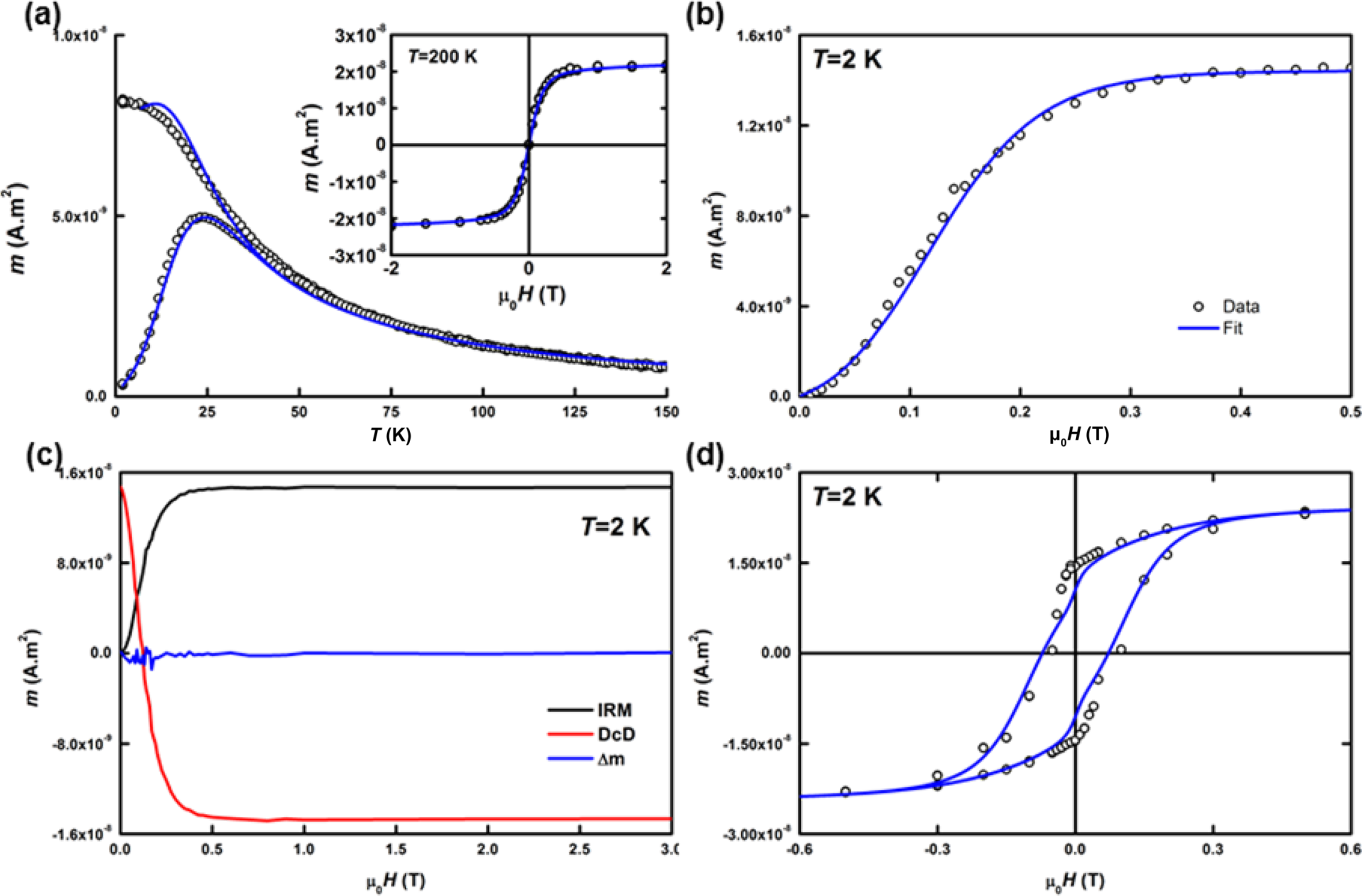
(a) ZFC/FC and *m*(*H*) experimental data for mass-selected as-prepared FeCo clusters with 4.3 nm in diameter along with their best fits; (b) IRM experimental data at 2 K with the corresponding biaxial contribution simulation; (c) IRM/DcD and δ*m* curves; (d) hysteresis loop at 2 K along with the corresponding simulation.

As illustrated in Figure 8a, the zero-field-cooling (ZFC) and field-cooling (FC) susceptibility curves show a transition from the superparamagnetic to the blocked regime for the as-prepared NPs with a maximum ZFC temperature (*T*_max_). *T*_max_ is connected to the energy barrier (Δ*E* = *K*_eff_*V*, with *K*_eff_ the effective anisotropy constant and *V* the magnetic volume) that a macrospin has to overcome to switch from one stable state to another one. We use our recently developed accurate “triple fit” method, where the ZFC/FC susceptibility curves and a high-temperature magnetization curve are fitted entirely simultaneously (Figure 8a). This simultaneous-fit protocol allows us to determine with good accuracy the parameters of our samples. Indeed, ZFC, FC and *m*(*H*) at high temperature can be expressed in a system of equations with common parameters, which are the total number of NPs, the magnetic diameter, the diameter dispersion, and the effective magnetic anisotropy constant. As demonstrated [21], only one set of parameters can fit the three curves at the same time. This “triple fit” method thus reduced the solution range of the different parameters and the uncertainty on their values. Alternating-current magnetic-susceptibility and ferromagnetic resonance measurements have not been used in our case because they are incompatible with the low quantities of matter in our samples. Then, we verified that the magnetic interactions are negligible in all our 1%-diluted samples. Based on the Wohlfarth relation [22], we can define the “well-known” parameter δ*m* [23–25] as follows:


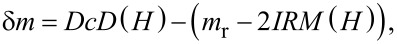


where *DcD*(*H*) is the direct-current demagnetization, *m*_r_ is the remanent magnetization and *IRM*(*H*) is the isothermal remanent demagnetization.

If there are dipolar interactions in the sample, the Henkel plot will not be a straight line as provided through the Stoner–Wohlfarth model. Thus, this deviation in the Henkel plot is related to the interaction between NPs. We verified this parameter for our samples and found it to be equal to 0 regardless of the applied magnetic field (see Figure 8b,c). The magnetization loop and the IRM curves at 2 K have been simulated with a modified Stoner–Wohlfarth model combined with the geometrical approach of the coherent rotation of magnetization [26]. We introduced a bi-axial contribution to completely describe the effective magnetic anisotropy energy (MAE) [27]. For all samples, we have reached a reliable determination of all the magnetic characteristic parameters such as the magnetic particle diameter *D*_m_ identical to that of the TEM distribution and the normal evolution of the *K*_eff_ distribution upon annealing.

**Figure 9:**
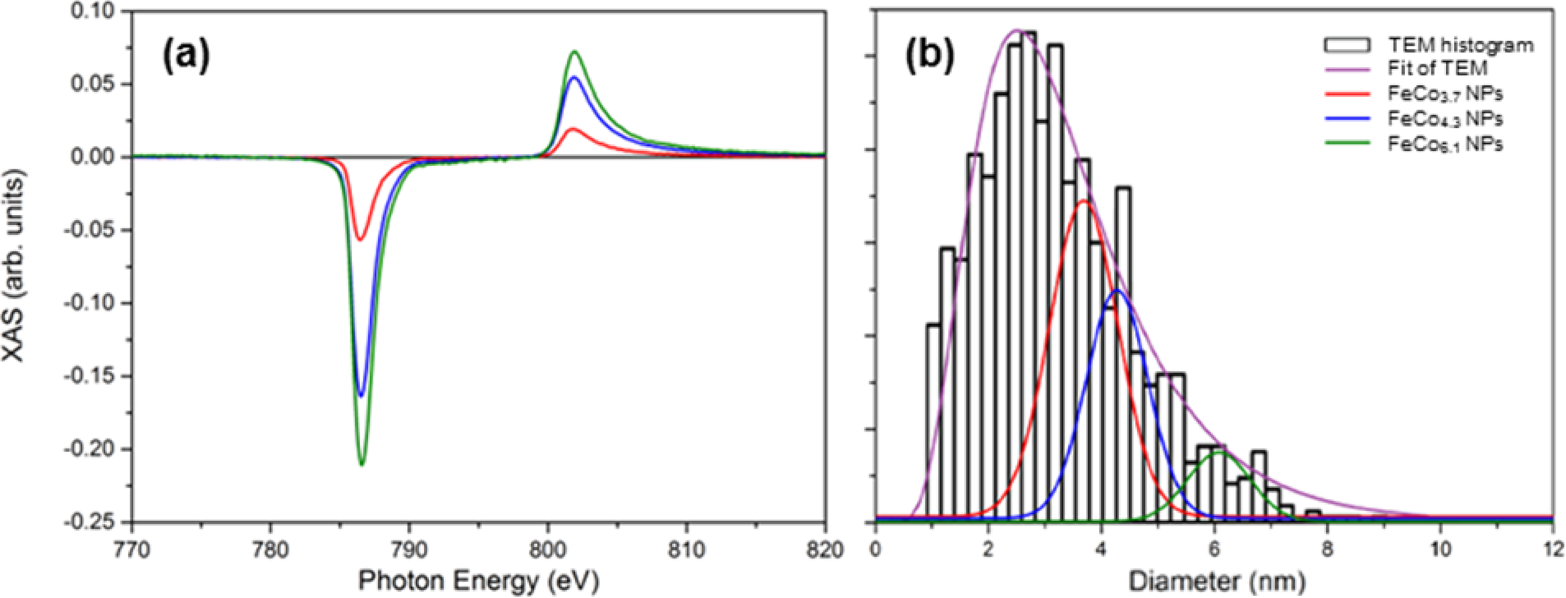
XMCD signal of as-prepared mass-selected FeCo samples at Co L_2,3_ edge (a) with their corresponding nominal diameter histogram compared to the whole TEM dispersion without selection (b).

**Figure 10:**
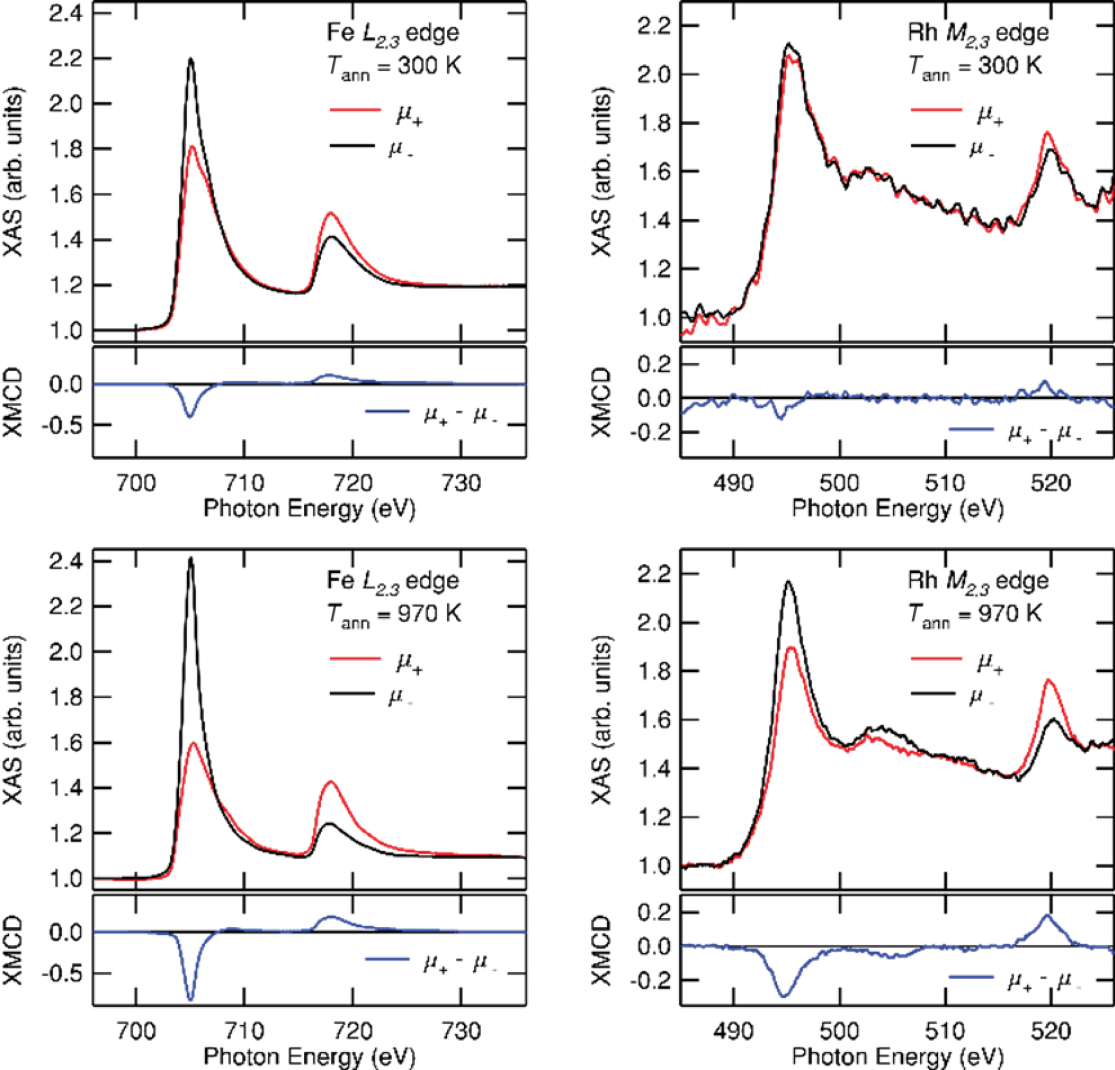
XMCD signals at Fe L_2,3_ edge (left) and at Rh M_2,3_ edge (right) measured at 3 K under 5 T before (top) and after annealing (bottom) on FeRh sample with 2 nm in diameter.

**Figure 11:**
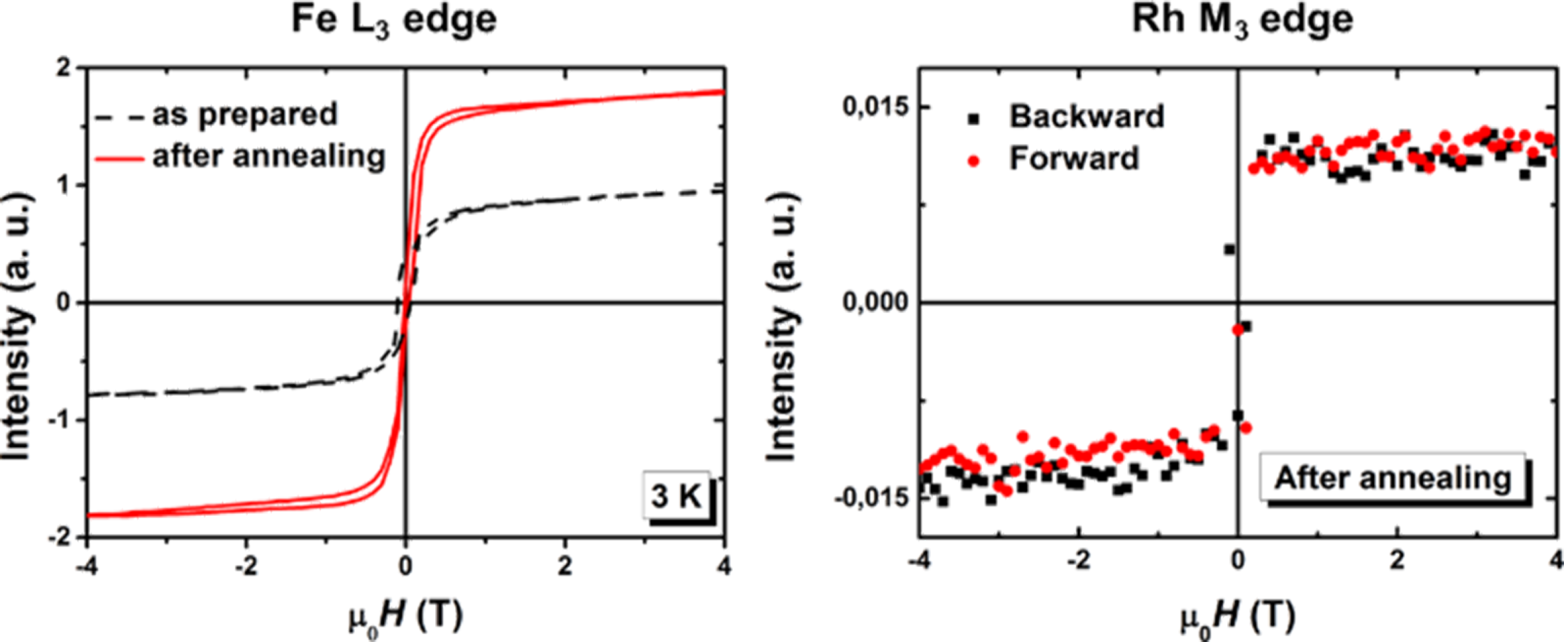
Magnetization curves obtained from XMCD signals measured at 3 K as a function of the applied magnetic field on as-prepared and annealed FeRh sample with 2 nm in diameter at Fe L_3_ edge (left) and only after annealing at Rh M_3_ edge (right).

In order to obtain the atomic magnetic moments and to correlate them to the finite-size effect in nanoalloys, we use XMCD spectroscopy experiments at each specific Fe and Rh M edge (respectively the Co L edge), at the “X-Treme” beamline at the Swiss Light Source for the FeRh sample and at the “DEIMOS” beamline at the SOLEIL synchrotron for FeCo for various nanocluster sizes (Figure 9); before and after annealing of the same sample at both edges (Figure 10 and Figure 11).

In the following section, we discuss the main results concerning the magnetic behaviour of the different samples. Notice that the MAE is mainly determined by semi-analytical models of all the SQUID magnetizations curves and that the spin and angular magnetic moments measured from XMCD are reached by using the sum rules [28–29].

## Discussion

At first, it is to notice that FeRh and FeCo samples are all ferromagnetic at low temperatures. In contrast to the anti-ferromagnetic order expected for the B2 phase in the bulk FeRh phase diagram (Figure 1a), we obtained the experimental persistence of high ferromagnetic magnetization down to 3 K for size-selected chemically ordered B2-like FeRh nanocrystals up to 5 nm in diameter. In particular, magnetic measurements on annealed 3.3 nm FeRh samples, have demonstrated ferromagnetic alignment of Fe and Rh at low temperatures with respective values of 3μ_B_ and 1μ_B_ [13]. The ferromagnetic order increases slightly for smaller nanoparticles as confirmed from XMCD measurements on annealed 2 nm FeRh (see Figure 10). It should be noted that the total magnetic moment of Rh which is expected to be non-magnetic in bulk, increases up to 1.5μ_B_ in 2 nm FeRh sample (see Figure 11). While it has long been known that the bcc FeRh unit cell volume expands upon transforming to the FM order [30], we use EXAFS experiments to determine the local iron environment before and after annealing. From a quantitative FEFFIT analysis at the Fe K edge on FeRh nanoparticle, we confirmed the systematic transition upon annealing from the chemically disordered fcc (A1) phase to the ordered CsCl-type (B2) structure for 3 nm FeRh clusters assemblies embedded in a carbon matrix [13]. In the latter case, the unit cell size has been found compatible with those of B2 FeRh bulk material with a Debye–Waller (DW) factor decreasing with chemical ordering. However, possibly due to relaxation effects at the nanoscale (as already observed in CoPt nanoalloys [31]), the DW parameter is still large upon annealing. This does not allow a perfect crystal with a homogeneous B2 structure, which would be expected to exhibit AFM magnetic order as in the bulk phase.

For FeCo, *T*_max_ is in the range of 10–50 K for as-prepared FeCo samples and increases upon annealing especially for larger magnetic diameters (*D* > 4 nm) in relation with a slight enhancement of *K*_eff_ [11]. For smaller sizes, MAE is rather constant (with *K*_eff_ ≈ 130 kJ/m^3^ as in FeRh NPs [10]). This is in agreement with the fact that MAE in NPs is dominated by the effect of additional facets with a large ω_K_ dispersion increasing with the number of possible chemical arrangements [32].

Contrary to what is expected for free 3D transition metal clusters [33] the magnetic signal of FeCo clusters increases with size for as-prepared and annealed FeCo clusters embedded in a carbon matrix. Moreover, the reduced magnetic moments in the FeCo nanoparticles, which could be due to the formation of a non-collinear structure at the interface with the matrix [34] remains below the threshold of 50% of the bulk average magnetic moment per atom. From a qualitative overview of the measured data on the annealed samples, we generally observed an enhancement of the spin and orbital moments at the Co L edge for the FeCo systems as expected for chemically ordered phase and metastable interface carbon demixing as observed in pure Co clusters [18]. Contrarily, at the Fe-L edge, especially for small size, the reduction of magnetic moment is probably related to a progression of non-magnetic stable iron carbide upon annealing. Further systematic analysis will be published in a near future on collected data on FeCo as well as both Fe and Co reference samples prepared by MS-LECBD [11].

## Conclusion

As a conclusion, we have obtained completely opposite thermal evolutions for the magnetic moments in CsCl-type (B2) chemically ordered FeRh and FeCo nanocrystal assemblies prepared by MS-LECBD. We have previously calculated that a Fe-based nanoparticle ranging from 2 to 5 nm in diameter (a few 100 to 6000 atoms per cluster) count, respectively, from 60% to 25% of the atoms at the interface between metallic atom cluster and carbon matrix atoms [10–11].

So on the one hand, because of the low chemical affinity of FeRh for carbon in the surrounding matrix, we have a strong proportion of relaxed first-neighbour distances at the surface of clusters in favour of FM order at low temperatures with uncompensated spins in the small FeRh nanoalloy, incompatible with AFM order at finite size. We are now preparing by the same MS LECBD technique, CsCl-type (B2) chemically ordered FeRh nanoparticles larger than 5 nm in diameter embedded in such inert carbon matrix, in order to determine the transition size for the temperature-dependent transition between FM to bulk-like AFM order.

On the other hand, even if some theoretical papers [35] predict that bulk FeCo alloys doped by carbon can lead to an enhanced magnetocrystalline anisotropy energy of up to 0.75 MJ/m^3^ by conserving 70% of the FeCo average magnetic moment per atom, we have shown that for FeCo nanoalloys in carbon environment especially for sizes smaller than 4 nm in diameter, the drawback of carbide formation is in competition with the benefit of tetragonal distortion expected for improving their magnetic properties. In this case, we have shown that the chemical reactivity of FeCo clusters with their environment can profoundly affects their structure and magnetic properties in complete contradiction with thermodynamic predictions [36].

More generally, the binary phase diagram and Néel/Curie temperature interplay, observed in bulk materials, still complicate the predictions of the size-dependent phase diagrams and magnetic behaviour in nanoalloys.
